# Syphilis and leprosy coinfection: A diagnostic conundrum

**DOI:** 10.1016/j.jdcr.2023.11.014

**Published:** 2023-11-25

**Authors:** María Alejandra Londoño-Echeverri, Fabio Samir Vargas-Cely, Jonny Alejandro García-Luna, Nelson Alberto Romero-Rosas, Liliana Eugenia Muñoz Garcia, Natalia Valderrama, Juan Carlos Salazar

**Affiliations:** a Escuela de Medicina, Universidad Libre, Cali, Colombia; b Centro Internacional de Entrenamiento e Investigaciones Médicas (CIDEIM), Cali, Colombia; c Universidad Icesi, Cali, Colombia; d Division of Dermatology, Department of Internal Medicine, School of Medicine, Universidad del Valle, Cali, Colombia; e Fundación Clínica Valle del Lili, Cali, Colombia; f Centro de Salud Panamericano, Programa Atención Enfermedad de Hansen, Cali, Colombia; g Department of Pediatrics, University of Connecticut School of Medicine, Farmington, Connecticut; h Division of Pediatric Infectious Diseases, Connecticut Children’s, Hartford, Connecticut; i Department of Immunology, University of Connecticut School of Medicine, Farmington, Connecticut

**Keywords:** leprosy, leprosy differential diagnosis, syphilis, syphilis serodiagnosis

## Introduction

Leprosy and syphilis cases are on the rise in some communities.^1^^,^^2^ Leprosy or Hansen disease is a neglected disease, classified by the World Health Organization as multibacillary or paucibacillary. It displays a wide spectrum of clinical phenotypes primarily involving the skin and peripheral nerves, ranging from macular lesions to disabling and mutilating disease. Its etiological agents are *Mycobacterium leprae* and *Mycobacterium lepromatosis*, which are obligate intracellular acid-fast bacilli.^3^ On the other hand, syphilis is a multistage chronic infection characterized by a long latent period following the initial symptomatic stages.^2^

An accurate diagnosis is essential, especially in tropical and subtropical contexts where multiple challenging and coexisting diagnosis should also be considered. Here, we describe the case of a woman with a chronic nodular dermatosis initially attributed to secondary syphilis but in whom a diagnosis of multibacillary leprosy coinfected with latent syphilis was established.

## Case report

A 39-year-old woman, from Cali, Colombia, was evaluated for a 6-month history of itchy macules on the limbs that evolved to red-brownish papules and nodules, some of them with mild scaling and ulceration. She also complained of dry skin, eyebrow hair loss and fatigue. She had no significant past medical history and was not receiving immunosuppressive medications. Due to concerns for secondary syphilis, her primary care physician performed a rapid plasma reagin (RPR) nontreponemal test (NTT), which was reactive at 1:128 dilutions, prompting her referral to our syphilis research clinic.

**Fig 1:**
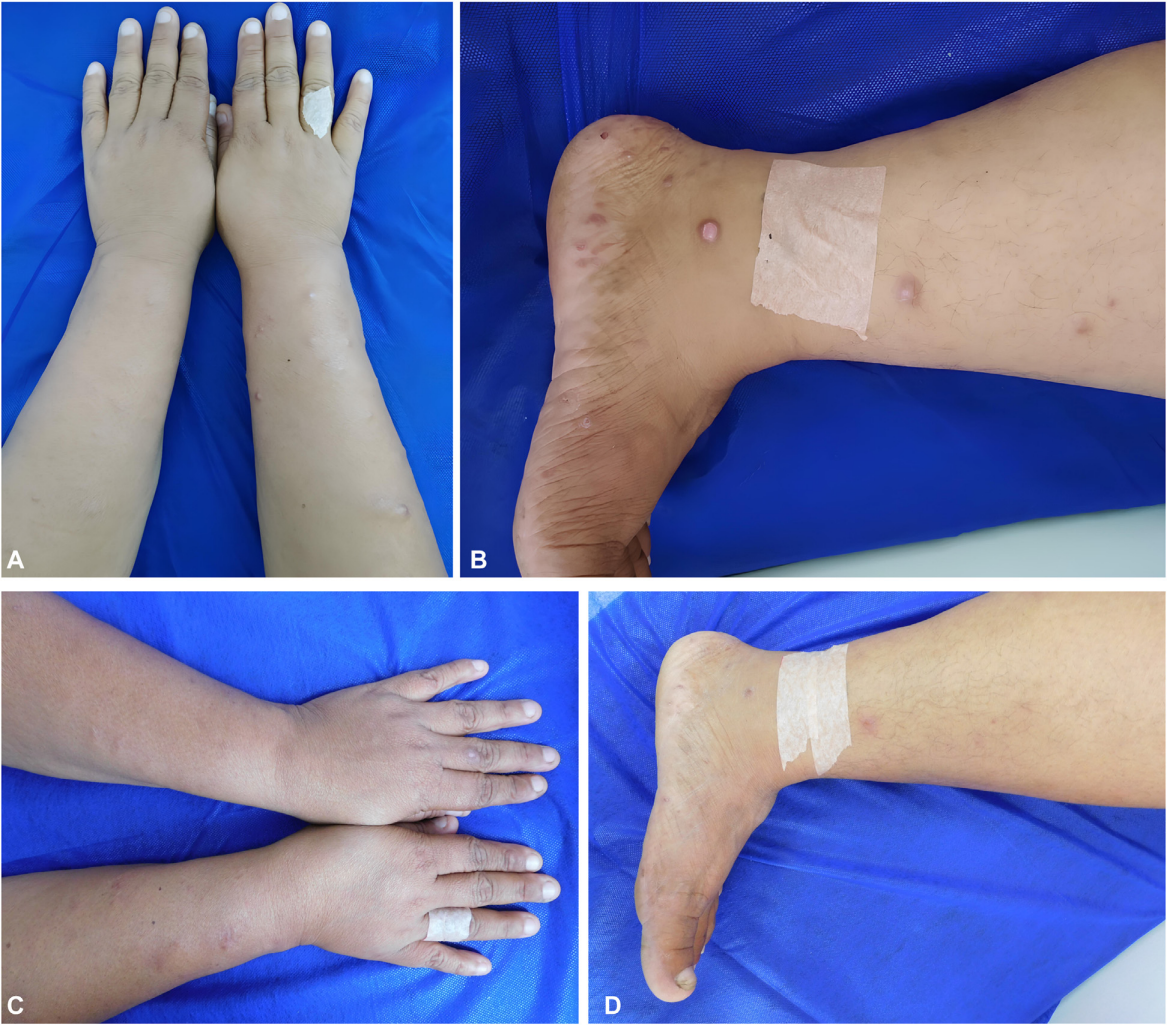
**A, B,** Skin findings during first visit. Erythematous nodules and papules on the upper and lower limb, some with superficial ulceration. **C, D,** Lesions aspect after 6 months of initiating treatment for leprosy. Notable clinical improvement with clearance of most elevated lesions, with some persistence of erythematous macules.

On initial physical examination, she displayed skin xerosis with multiple red-brown nodules over the forearms, both ankles and feet, some with mild scaling and superficial ulceration (Fig 1, *A*, *B*). There were no signs of cutaneous sensory loss on or near the skin lesions. Repeated blood work performed at a reference laboratory, 2 days after the initial RPR, reported a result of 1:2 dilutions, with a reactive confirmatory chemiluminescent magnetic micro particle immunoassay treponemal test (TT). A fourth generation enzyme-linked immunosorbent assay for HIV was negative. Hepatitis B and C were ruled out and hematologic parameters were normal. An initial diagnosis of nodular secondary syphilis was considered, and treatment was provided with 3 doses of 2,400,000 U of benzathine penicillin G (1 dose weekly) per local treatment guidelines.

**Fig 2:**
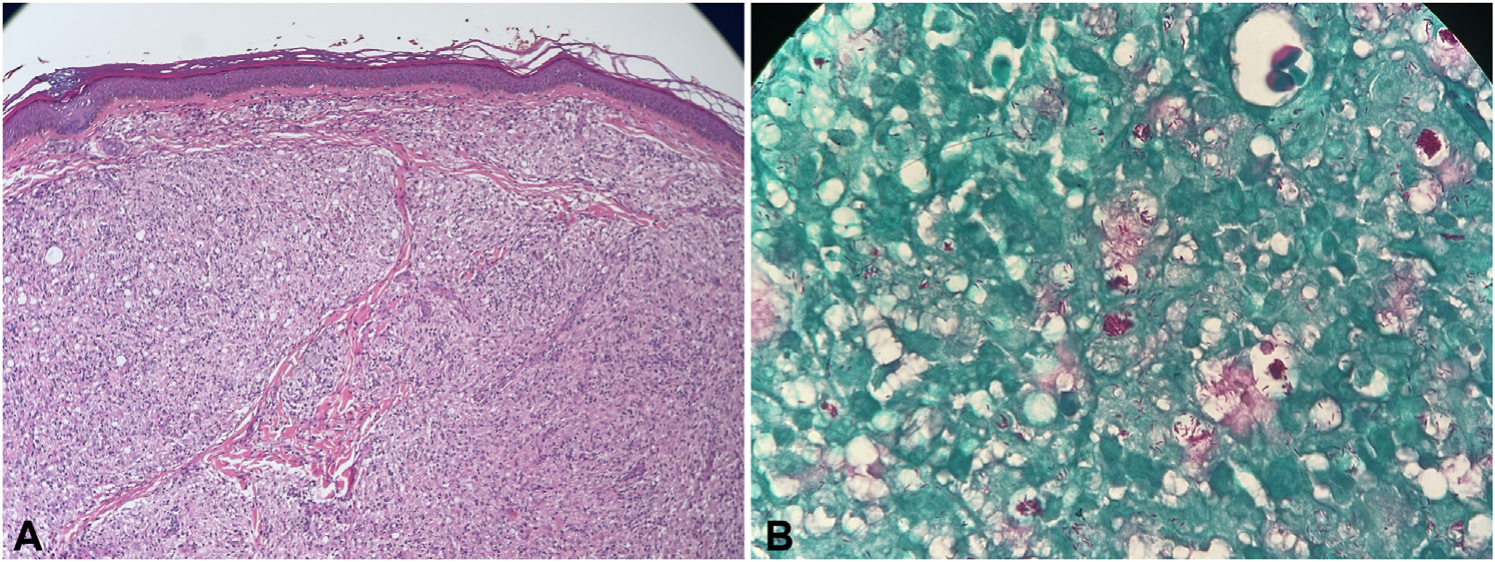
Forearm nodule. **A,** Hematoxylin and eosin staining (10× magnification) showing chronic granulomatous inflammation with abundant lymphohistiocytic infiltrate and vacuolated cells in the dermis, separated from the epidermis by a normal collagen band known as Unna’s band typical of lepromatous leprosy. **B,** Fite-Faraco staining (100× magnification) depicting abundant individual and grouped (globi) acid-fast bacilli. Photos courtesy of Dr Ricardo Rueda, Department of Pathology, Universidad del Valle.

At 6-month follow-up and despite adherence to syphilis treatment, the patient did not show any clinical improvement. The posttreatment RPR, performed in the same reference laboratory, yielded the same titer of 1:2 dilutions. Given the lack of clinical response to treatment for syphilis, we considered alternative diagnosis and performed excisional biopsy of 2 nodules. Histopathology study (Fig 2, *A*) revealed chronic granulomatous inflammation with globi grouped acid-fast bacilli on Fite-Faraco staining (Fig 2, *B*), confirming a diagnosis of lepromatous leprosy (multibacillary). *Treponema pallidum* DNA was not identified in the biopsy by polymerase chain reaction *PolA* gene amplification.^4^

Considering the inconsistent RPR results between the primary health center and our reference laboratory, we considered the initial RPR as falsely elevated and established the diagnosis of latent syphilis of unknown duration based on the positive TT, the reference RPR titer of 1:2 and the lack of previous history of syphilis diagnosis or treatment.

**Fig 3:**
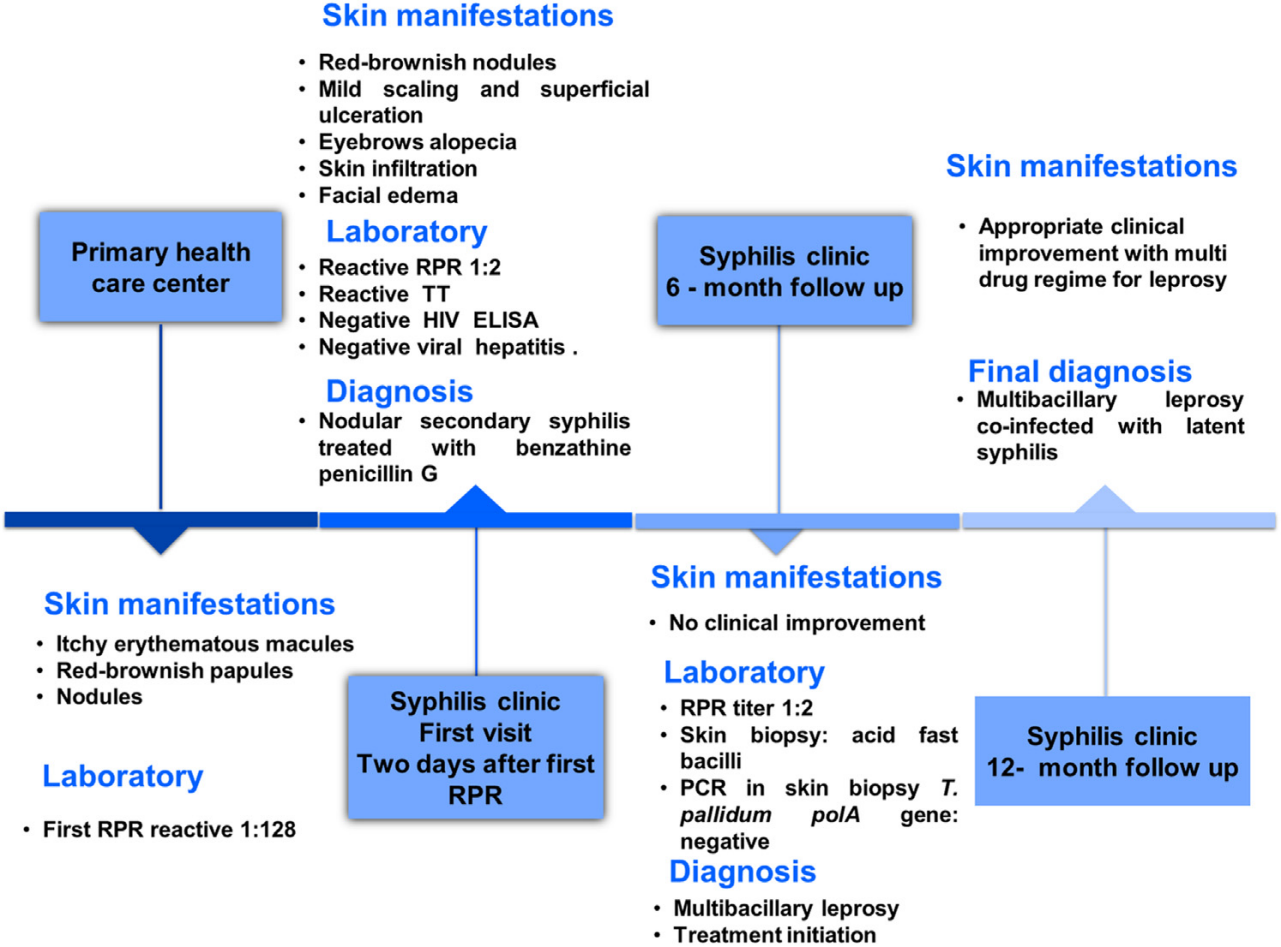
Summary timeline of clinical events. *ELISA*, Enzyme-linked immunosorbent assay; *HIV*, human immunodeficiency virus; *PCR*, polymerase chain reaction; *RPR*, rapid plasma reagin; *TT*, treponemal test.

The patient was initiated on a multidrug regimen for Leprosy according to World Health Organization guidelines (rifampicin 600 mg monthly; dapsone: 100 mg daily and clofazimine: 300 mg once a month and 50 mg daily during 12-months). At 6-month follow-up the patient was found to be markedly improved (Fig 1, *C*, *D*). For timeline details see Fig 3.

## Discussion

Leprosy is a chronic infectious disease with >200,000 cases reported every year^5^ with the highest burden of disease held by the Americas, Africa, and South-East Asia. Decreased clinical awareness, socio-geographic isolation of endemic rural regions and a challenging clinical diagnosis, especially in the initial stages of disease contribute to misdiagnosis, diagnostic delay and disease progression.^6^ It can manifest with variable degrees of skin and peripheral nerve compromise causing clinical features that can resemble alternative differential diagnoses such as deep mycosis, autoimmune diseases and syphilis, among others.^6^

In this case the documentation of a positive TT and a falsely elevated NTT suggested this patient had nodular syphilis. However, this form of syphilis is unusual in HIV-negative individuals and the distribution of lesions tend to predominate in the head and neck, which is the opposite location of lesions described in this case.^7^ Furthermore, the finding of a discordant pretreatment RPR and lack of clinical improvement following penicillin therapy, led us to revisit our initial impression and confirm the diagnosis of lepromatous leprosy by histopathology. This case highlights the importance of considering alternative diagnosis based on clinical outcomes of initial interventions.

The differential diagnosis for syphilis and leprosy is extensive. Both diseases can resemble each other^8^ and can present as coinfections.^9^ NTT for syphilis have many pitfalls contributing to potential misdiagnosis including cross-reactivity with antibodies produced in other infections (endemic treponematoses, HIV, viral hepatitis, and leprosy), inflammatory conditions and plasma cell diseases. The presence of a positive specific TT and the lack of previously diagnosed or treated syphilis episodes suggested a true positive case of syphilis and elicited the diagnosis of latent syphilis of unknown duration, which is an asymptomatic stage with usually low NTT titers.^2^

Differences higher than 2 dilutions in NTT between reference and primary health care laboratories are not uncommon in low and middle income settings.^10^ This variability causes additional challenges in the diagnosis of syphilis,^10^ especially when previous NTT results are not available, as in our case. In clinical practice, treatment should be warranted for both diseases, even for those cases where cross-reactivity in NTT is suspected, given the risk-benefit favoring the administration of penicillin in false positive syphilis against not treating a true positive syphilis.

In conclusion, this challenging case highlights the diagnostic dilemmas evoked by skin dermatosis especially in the tropics, where both syphilis and leprosy coexist in the community. Clinicians need to be well versed in the identification of both diseases and must be ready to perform skin biopsies, as was done in this case, when the diagnosis is unclear.

## Conflicts of interest

Dr García-Luna has received support from Janssen, Carnott, Epidermique, and Cantabria laboratories to attend dermatology meetings in Colombia, outside the scope of this work. Drs Londoño-Echeverri, Vargas-Cely, Romero-Rosas, Muñoz Garcia, Valderrama and Salazar have no conflicts of interest to declare.
